# A Liposomal Formulation Able to Incorporate a High Content of Paclitaxel and Exert Promising Anticancer Effect

**DOI:** 10.1155/2011/629234

**Published:** 2010-10-11

**Authors:** Pei Kan, Chih-Wan Tsao, Ae-June Wang, Wu-Chou Su, Hsiang-Fa Liang

**Affiliations:** ^1^Drug Delivery Lab, Biomedical Engineering Research Laboratories, Industrial Technology Research Institute, Hsinchu 31040, Taiwan; ^2^Medical College and Hospital, National Cheng Kung University, Tainan 701, Taiwan

## Abstract

A liposome formulation for paclitaxel was developed in this study. The liposomes, composed of naturally unsaturated and hydrogenated phosphatidylcholines, with significant phase transition temperature difference, were prepared and characterized. The liposomes exhibited a high content of paclitaxel, which was incorporated within the segregated microdomains coexisting on phospholipid bilayer of liposomes. As much as 15% paclitaxel to phospholipid molar ratio were attained without precipitates observed during preparation. In addition, the liposomes remained stable in liquid form at 4°C for at least 6 months. The special composition of liposomal membrane which could reduce paclitaxel aggregation could account for such a capacity and stability. The cytotoxicity of prepared paclitaxel liposomes on the colon cancer C-26 cell culture was comparable to Taxol. Acute toxicity test revealed that LD_50_ for intravenous bolus injection in mice exceeded by 40 mg/kg. In antitumor efficacy study, the prepared liposomal paclitaxel demonstrated the increase in the efficacy against human cancer in animal model. Taken together, the novel formulated liposomes can incorporate high content of paclitaxel, remaining stable for long-term storage. These animal data also demonstrate that the liposomal paclitaxel is promising for further clinical use.

## 1. Introduction

 Paclitaxel, an effective anticancer agent, has been applied as the first-line drug against breast and ovarian cancers. However, more extensive clinical use is limited owing to the drug's low water solubility and the highly inflammatory response to the current excipient, cremophore EL [1]. Thus, much effort has been made in eliminating the side effects during administration. A variety of formulations have been developed to replace cremophore EL [2–12]. Among those formulations, liposome is regarded as one of the most promising drug carrier. It has many advantages over other formulations, such as being the most biocompatible and best able to reduce drug toxicity without changing drug efficacy against tumor cells. However, limited drug loading and insufficient shelf stability remain prohibitive obstacles to practical application [1, 2, 12]. 

 Conventional paclitaxel liposomes were prepared at a confined paclitaxel/lipid molar ratio of approximately 3%, regardless of whether the liposomes were made of a mixture of phosphatidyl glycerol (PG) [13, 14] or DOTAP (1,2-dioleoyl-3-trimethylammonium propane) [15] and phosphatidyl choline (PC), or unsaturated [16, 17] or partially unsaturated PC [18]. At a drug-to-lipid molar ratio of 4%, the paclitaxel-liposomes are stable for only 2 days. During preparation of paclitaxel liposomes, needle-like crystal precipitates appear at a drug/lipid molar ratio up to 8% [19]. Incorporation of the hydrophilic polymer conjugated phospholipid (methoxy polyethylene glycol-phosphatidyl ethanolamine), known to be able to stabilize liposomes and extend its circulation time in the bloodstream, that was attempted [20]. But the PEGylated liposomes with a maximal paclitaxel/lipid molar ratio of 3% quickly become unstable in one week of storage at 4°C. On the other hand, the liposomal formulations of paclitaxel consisting of a special negatively charged phospholipid, cardiolipid, and phosphatidyl choline have been described [21]. Increasing the paclitaxel/lipid molar ratio to 9% causes the liposomes to be stable for only one month when stored in liquid form at 4°C [21]. For the 3% drug-to-lipid ratio of liposomal paclitaxel, it needs many lipids to formulate, thus increases the cost of lipids and the volume for injection in clinical, and would be significantly more expensive than the commercial product (Taxol).

 Korlach et al. reported the presence of a phase separation in giant unilamellar vesicles composed of DPPC/DLPC/cholesterol was visualized [22]. It was speculated that there are many segregated microdomains coexisting on the membrane of liposomes constituted by two different kinds of lipids. We hypothesized these microdomains might prevent the aggregation of hydrophobic drug to form crystal precipitates.

 The aim of the study was to develop a novel liposomal formulation, composed of naturally unsaturated and hydrogenated PC with significant phase transition temperature difference, capable of incorporating high paclitaxel content. The influences of the feeding ratio of hydrogenated PC to total PC and the drug-to-lipid ratio on the particles size, drug incorporation efficiency, phase transition temperature, and the storage stability were evaluated. Additionally, *in vitro* cytotoxicity of prepared paclitaxel-loaded liposomes on C-26 colon cancer cell line was estimated. Moreover, plasma exposure and acute toxicity of the paclitaxel liposomes were studied *in vivo*. Finally, the antitumor efficacy of the paclitaxel liposomes in PC14PE6/AS2 bearing nude mice was also examined.

## 2. Materials and Methods

### 2.1. Materials

Egg phosphatidylcholine (EPC, Lipoid E100), and hydrogenated egg phosphatidylcholine (HEPC, Lipoid E PC-3) were obtained from Lipoid GmbH. Hydrogenated soy phosphatidylcholine (HSPC, Epikuron 200 SH) were obtained from Lucas Meyer GmbH. Paclitaxel was purchased from Hauser Chemical Res, Inc. Methoxy polyethylene glycol 2000-disteary phosphatidyl ethanolamine (MPEG) was purchased from Shearwater Polymers, Inc. The other chemicals were purchased from Sigma or Merck.

### 2.2. Preparation of Liposomes

 Paclitaxel was added to the alcoholic admixture of EPC, HEPC, cholesterol (Chol), and MPEG with a given drug-to-lipid molar ratio as indicated in the context. The solution was evaporated under vacuum to remove the solvent and formed a lipid film on the wall of the round-bottom flask at which time; aliquots of 10% (w/v) sucrose were added to the flask for hydration. Large multilamellar liposomes were suspended, and then sonicated (XL2020, Misonix Inc., Farmingdale, NY, USA) for 10 minutes to yield small unilamellar liposomes. Paclitaxel-containing liposomes underwent filtration through a 0.2 *μ*m cellulose acetate membrane (Orange Scientific Co., Braine L' Alleud, Belgium) to remove possible paclitaxel precipitates and achieve sterilization. Drug incorporation efficiency, representing the retention of paclitaxel in the filtered liposomes with respect to the originally added drug, was determined by HPLC analysis. Laser particle size analyzer (N4 Plus, Coulter Electronics Inc., Hialeah, FL, USA) was used to measure the average particle size. The liposomes were sealed in the vial under nitrogen and stored at 4°C for further shelf stability test.

### 2.3. HPLC Assay

 High performance liquid chromatography (HPLC) was performed using an autosampler, controller, and dual wavelength absorbance detector with wavelength set to 229 nm, all of which were obtained from Waters Co. A 125 mm × 4 mm Lichrosphere 100 RP-18 column, obtained from Merck, was employed to identify and quantify the concentration of paclitaxel. The mobile phase was composed of 50% acetonitrile and 50% D.I. water eluted isocratically throughout the measurement. A sample was dissolved in methanol before injection into a 20 *μ*L sample loop. The retention time of paclitaxel is 12 minute while the flow rate was kept at 0.5 mL/min.

### 2.4. Differential Scanning Calorimetry (DSC) Studies

 DSC measurements were performed using a differential scanning calorimeter (Mettler-Toledo DSC 822e, Switzerland). The liposome suspensions (total lipid concentration: 20 mg/mL) were heated at a programmed constant heating rate of 5°C per minute. Empty hermetically sealed aluminum pans were used as reference.

### 2.5. Shelf Stability Analysis

 Shelf stability of the paclitaxel liposomes at 4°C was monitored at the predetermined interval time. Particle size was analyzed before filtration of the sample to remove the aggregated liposomes and paclitaxel precipitates. The sample filtered through 0.2 *μ*m cellulose acetate membrane then was prepared for measurement of paclitaxel concentration by HPLC.

### 2.6. Cytotoxicity Assay

 C-26, a syngeneic colon tumor cell line, was inoculated at 5 × 10^3^ cells per well in 96-well microtiter plate. The cells were maintained with RPMI-1640 medium comprising 10% heat-inactivated fetal calf serum, 100 U/mL penicillin, and 100 mg/mL streptomycin at 37°C in a 5% CO_2_ humidified incubator. The drug-containing solutions were added and incubated with cells for 72 hours before the MTT assay [23]. Liposome vehicle at the comparable lipid concentration was used as the control. The optical density readings were determined by an ELISA reader at 540 nm. Cell survival rate was calculated by internalization of the optical density readings.

### 2.7. Pharmacokinetic Studies

 All animal studies, including pharmacokinetic study, acute toxicity, and efficacies of prepared liposomes, were performed in compliance with the “Guide for the Care and Use of Laboratory Animals” prepared by the Institute of Laboratory Animal Resources, National Research Council, USA and published by the National Academy Press, revised in 1996.

 For the pharmacokinetic studies, six to seven weeks old female SD rats were purchased from the National Laboratories of Animal Breeding and Research Center (NLABRC, Nangarng, Taipei, Taiwan). Rats were bred at least one week after received to obtain a stable habitable condition before any experiment. The jugular vein was cannulated and the cannula was exteriorized in the back of the neck. Taxol or liposomal paclitaxel was administrated through the jugular vein at the paclitaxel dose of 5 mg/kg rats. Serial blood samples were withdrawn through the venous catheter after the rats were awakened from anesthesia. Drug concentrations in plasma were analyzed by HPLC. The pharmacokinetic parameters of each formulation were calculated using the WinNonLin pharmacokinetic software (Version 3.1, Pharsight Co., Mountainview, CA, USA).

### 2.8. Acute Toxicity Test

Six to eight weeks old male ICR mice were divided into four different groups (treated with Taxol 20 mg/kg, Taxol 40 mg/kg, liposomal paclitaxel 20 mg/kg, or liposomal paclitaxel 40 mg/kg), consisting of 5 mice in each group. Mice for each group were injected through tail vein to examine the acute intravenous toxicity. After liposome administration, the mice were observed for 14 days. During the observation period, mice were observed daily for mortality and clinical signs. The survival rate over 14 days was obtained.

### 2.9. Efficacy Test

 Athymic BALB/c nude mice were obtained from NLABRC and weighted 20–22 g at the start of the experiments. The mice were housed in sterilized filter-topped cages and maintained in sterile conditions. The human lung adenocarcinoma cell line PC14PE6/AS2, a derivative of PC14PE6, which was obtained from Dr. Wu-Chou Su (National Chung Kung University medical college, Taiwan). The PC14PE6/AS2 cells express higher VEGF proteins, microvessel density, and vascular permeability in tumors [24]. It was suggested that the enhanced permeability and retention (EPR) effect within the tumor site made colloidal systems more effective on the treatment of cancer [25]. On the day of implantation, 10^6^ cells were inoculated subcutaneously into lower back for each mouse. Tumor volume was determined by measuring orthogonal diameters of the tumor and calculated as 0.4 × (*a* ^2^ × *b*), where “*a*” is the tumor width and “*b*” is its length in mm. Tumor formation measuring at least 250 mm^3^ was considered a positive take (day 0), at which time 4 groups, each contained 6 animals, were established. They were (1) normal saline control group, (2) Taxol 20 mg/kg treatment group, (3) liposomal paclitaxel 20 mg/kg treatment group, and (4) liposomal paclitaxel 40 mg/kg treatment group. The drugs and controls were given as a bolus into tail veins for 4 doses totally on day 1, 3, 6, and 9. Animal mortality was checked daily, and tumor volume and body weight was checked every other day. Mice showing more than 20% body weight loss or tumors larger than 10-fold of original size (~2,500 mm^3^) were sacrificed.

## 3. Results

### 3.1. Liposomes Made of Single PC

 The liposomes composed of either HEPC or EPC alone were prepared according to the procedure described in Experimental Section. MPEG was used in the formulation to stabilize liposomes. The MPEG to phospholipid molar ratio was limited to less than 5% to avoid misinterpretation with the combinative formulation [26]. The cholesterol compositions were optimized to obtain the small liposome size and high drug incorporation. The results in Table 1 showed that the liposomes made mainly of EPC incorporated up to 88% paclitaxel when the drug to phospholipid molar ratio was kept at 3%. However, drug incorporation efficiency fell to 42% when the drug to phospholipid molar ratio rose to 7%. The liposomes made of HEPC incorporated below 70% paclitaxel when drug to phospholipid molar ratio was kept at 3%. The liposomes would yield apparent white precipitates while paclitaxel to phospholipid molar ratio was elevated above 3%. The higher drug incorporation efficiency for EPC liposomes resulted from the lower transition temperature of EPC (<0°C) that is flexible enough to entrap relatively more hydrophobic molecules rather than rigid HPEC [27]. Table 1 also presents the shelf stability of the liposomes composed principally of either HEPC or EPC. These liposomes were monitored at 4°C only for one month because of the early appearance of white precipitates. A decrease in drug incorporation efficiency in the liposomes was confirmed by HPLC. The EPC liposomes exhibited an obvious decline in drug incorporation as the drug to phospholipid molar ratio was increased to 7%. The HEPC liposomes were considerably unstable too.

### 3.2. Combinative Formulation of Hydrogenated PC and Natural PC

 Two distinct phosphatidylcholines with significant phase transition temperature difference were used in this study. HEPC is referred to as a phospholipid with high phase transition temperature, anticipated to be about 50–60°C. Natural EPC containing high content of unsaturated fatty acid chains is considered to have low-phase transition temperature below 0°C. A series of combinations of HEPC and EPC were investigated in an attempt to develop a stable liposome formulation for paclitaxel. Besides, 5 mol% MPEG and 10 mol% cholesterol to phospholipid molar ratio were added to costabilize the liposomes. Their effects of MPEG and cholesterol on paclitaxel incorporation and particle size were minimized by the constant molar ratio. The characteristics of the formulated liposomes are given in Table 2. When increasing HEPC molar ratio, this led to an increase in the average diameter of the liposomes. Meanwhile, the incorporation efficiency of paclitaxel gradually decreased as the quantity of HEPC increased. The particle size could be reduced but drug incorporation efficiency did not change significantly when HEPC molar ratio decreased below 62%. Therefore, 25% molar ratio of HEPC, with the smallest particle size among the tested compositions, was selected to test the drug loading capacity of the liposome formulation.

### 3.3. Increasing Drug/Phospholipid Ratio

 Drug loading capacity of the formulated liposomes described above was investigated by further increasing paclitaxel to phospholipid molar ratio from 7% to 25%. Phospholipids represent HEPC, EPC, and MPEG. Drug to phospholipid molar ratio represents the originally added drug content. The effects of increasing drug to phospholipid ratio were examined on the physical properties of drug incorporation and particle size. Table 3 lists the drug incorporation efficiency and particle size of the resultant liposomes. It was noteworthy that high paclitaxel content was effectively incorporated in the liposomes. The incorporation efficiency was maintained above 80% even though the drug to phospholipid molar ratio was increased to 20%. No precipitate was observed throughout preparation.

 The drug loading capacity of the liposomes was found to be paclitaxel concentration dependent. Attempts to incorporate extremely high paclitaxel tended to destabilize liposomes. The drug incorporation efficiency dropped to 55% when the drug to phospholipid molar ratio was increased to 25%. Such a high paclitaxel loading accelerated destabilization of liposomes. White precipitates and aggregated liposomes appeared shortly after sonication. Needle-like precipitates and many floccules can be seen by optical microscope. The poor drug incorporation and lipid aggregation reflects instability of the liposomes with such a high drug to phospholipid ratio. Therefore, the maximum drug loading in the stable liposomes during preparation was anticipated to 20 mole%. Besides, liposome solutions with various lipid concentrations were also prepared and examined. It is evident that doubling lipid content (40 mM) with the same liposome composition affected neither drug incorporation nor particle size.

### 3.4. Phase Transition Temperature of Prepared Liposomes

**Figure 1:**
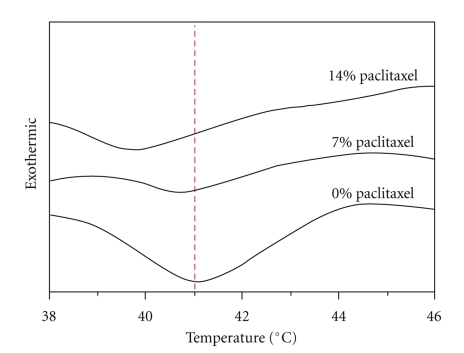
The DSC thermographs for the EPC/HEPC (4 : 1) liposomes without the drug as well as with 7 and 14 mol% paclitaxel.

 To determine the influence of paclitaxel on phospholipid bilayer phase transitions, the DSC analysis was employed. The DSC thermographs for the EPC/HEPC (4 : 1) liposomes without the drug as well as with 7 and 14 mol% paclitaxel are shown in Figure 1. For the EPC/HEPC liposome, a low miscibility was observed that leads to a phase separation in the temperature range of 39–44°C. It can be observed from Figure 1 that with increasing the paclitaxel concentration in liposomes, the main transition temperature was shifted slightly to a lower temperature from 41°C to 39.5°C.

### 3.5. Shelf Stability

**Figure 2:**
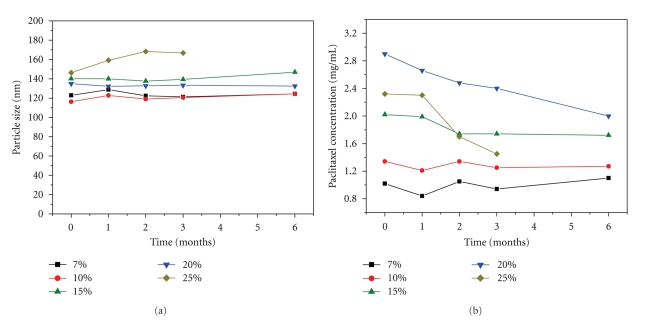
Shelf stability of the liposomes with increasing drug/phospholipid molar ratio at 4°C. Legends mean the paclitaxel to phospholipid molar ratio of the liposomes. The composition is tabulated in Table 2.

 Liposomal paclitaxel were stored at 4°C immediately after preparation and sterilization. Particle size (Figure 2(a)) and paclitaxel concentration (Figure 2(b)) were measured periodically. The results in Figure 2 indicate that these two measurements were stable for most of the formulations over six months. The implication of shelf stability of the liposomes with paclitaxel to lipid ratio was revealed. At 25% of drug to phospholipid molar ratio the liposomes was rather unstable in both terms of drug incorporation and particle size. Particle size rose and drug incorporation fell apparently. Although the 20% liposomes possessed more than 80% of incorporation efficiency at the beginning of storage, drug incorporation declined much more and faster than those of the other with a lower drug-to-lipid ratio. After six-month storage, the retention of paclitaxel in the liposomes dropped to 67% of the originally incorporated amount. The particle size increased from 146 to 168 nm within the first two months. When the drug to phospholipid molar ratio was maintained equal or below to 15%, all the formulated liposomes remained stable for at least 6 months. The particle size varied by 10 nm in maximum and almost unchanged drug incorporation occurred. A maximum drug loading capacity of the liposomes, which could be stable in long-term storage, thus was anticipated to be 15%–20% drug-to-lipid molar ratio.

### 3.6. Cytotoxicity

**Figure 3:**
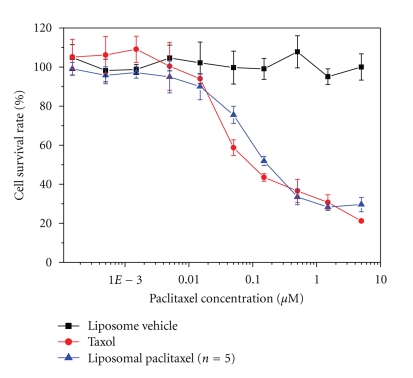
Survival rates of C-26 tumor cells exposing to the liposomes with or without paclitaxel and Taxol. The amount of the liposomes corresponding to the paclitaxel liposome was added as the control.

 The liposome formula with 15% paclitaxel was preceded with the cytotoxicity, acute toxicity, and pharmacokinetic tests. The paclitaxel concentration of the liposomes for 50% inhibition of C-26 cells (IC_50_) is approximately 162 nM which is slightly higher than that of Taxol (IC_50_ = 105 nM), as shown in Figure 3. The liposome vehicles without paclitaxel showed no cytotoxicity against C-26 tumor cells over the tested range.

### 3.7. Pharmacokinetic Studies

**Figure 4:**
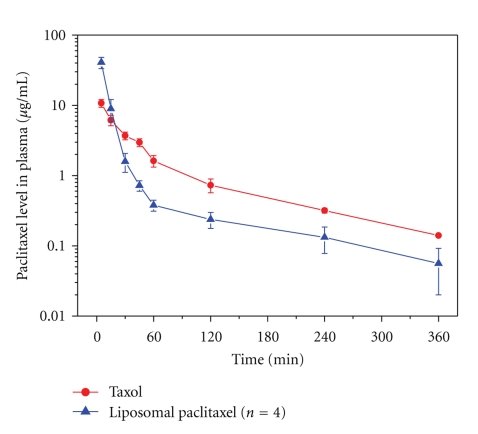
Plasma concentration profiles of paclitaxel after i.v. injection of Taxol or paclitaxel liposomes in rats (5 mg/kg as paclitaxel). Each data represents the mean of 4 rats.

Figure 4 and Table 4 show the plasma concentration profile of paclitaxel and their pharmacokinetic parameters, respectively, after i.v. injection of liposomes and Taxol in rats. The AUC value of paclitaxel liposomes was slightly higher than that of Taxol. However, the liposomal paclitaxel in plasma declined quicker than Taxol. It seems that incorporation of MPEG in the prepared liposome formulations did not prolong their circulation time.

### 3.8. Acute Toxicity

 Escalated dose of paclitaxel liposomes was tested in ICR mice to determine the acute toxicity. Mice were divided into two groups treated with Taxol or liposomal paclitaxel at doses of 20 and 40 mg/kg, respectively. Table 5 shows the survival rate in all the groups over 14 days. It was found that 3 of 5 mice with 40 mg/kg of Taxol group died on the same day of injection. Afterwards, one of the other mice died on the third day. In contrast, all the mice in liposomal paclitaxel group survived over the test period of 14 days.

### 3.9. Efficacy Test

**Figure 5:**
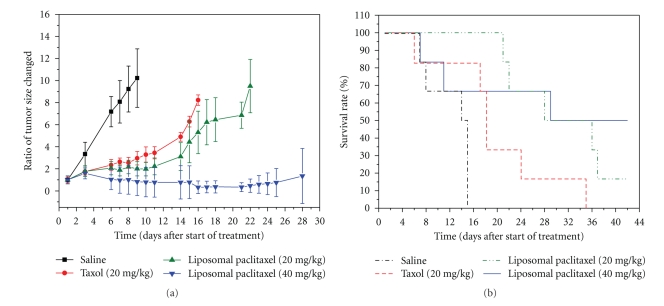
(a) The ratio of tumor size changed and (b) survival rate of different paclitaxel formulations on human lung adenocarcinoma (AS-2) bearing nude mice.

 The antitumor efficacy of distinct paclitaxel formulations was studied in AS-2 lung cancer bearing nude mice. In the case of the paclitaxel liposomes, weight loss was observed at a dose of 40 mg/kg. One mouse died on day 7 and one on day 11. It was estimated the toxicity due to the repeated doses of the paclitaxel liposomes. In the case of Taxol at a dose of 20 mg/kg, an evidence that one mouse died on day 6 also indicated the repeated dose toxicity. Because Taxol at a dose of 40 mg/kg had caused a high mortality in nude mice (>50%) in a preliminary study, we excluded the dose level in the current study.  Figure 5(a) shows the progress of the tumor growth observed for 28 days. It was found that the tumor size of the normal saline group increased significantly with time. In contrast, the groups injected with distinct paclitaxel formulations significantly delayed the tumor growth as compared to the normal saline group (*P* < .05). At the same dose of 20 mg/kg, liposomal paclitaxel seemed to delay the tumor growth more effectively than Taxol. Once increasing the dose to 40 mg/kg, the liposomal paclitaxel significantly inhibit the tumor growth for more than 42 days as compared with other treated groups (*P* < .05). Although two mice died during dosing treatment for the high dose of liposomal paclitaxel, the liposomal paclitaxel (20 and 40 mg/kg) significantly enhanced the mouse survival time to more than 30 days as compared with saline group (Figure 5(b), *P* < .05). The median survival time for mice treated with normal saline was 12.3 days, and treatment with Taxol slightly increased this survival to 19.7 days. Thus, the prepared liposomal paclitaxel provide benefits on reducing tumor volume, which correlated with a substantial increase in animal survival.

## 4. Discussion

 Balasubramanian et al. reported that paclitaxel has a tendency to undergo concentration-dependent aggregation in hydrophobic or relatively low polarity environments, forming intermolecular hydrogen bonds [28]. Restated, as a large amount of paclitaxel is embedded in the hydrophobic domain within the bilayer membrane, it is thermodynamically prone to self-aggregating, and thereby destabilizing the liposomes [29]. The results imply the limited drug loading and the poor shelf stability of the current liposome formulations for paclitaxel. Much research [12–15, 19] also supported the fact that the optimal paclitaxel to lipid molar ratio in the previous liposome formulations is from 3% to 4%, and the liposomes is shelf stable only when the drug-to-lipid molar ratio is kept equal to or below 3%. A higher drug-to-lipid molar ratio would lead to the occurrence of needle-like crystal precipitate during preparation.

 To improve instability and poor drug payload of the conventional paclitaxel liposomes, we developed a formulation combining two sorts of PCs into liposomes, which have significant differences between their phase transition temperatures. Based on the material information provided by the manufacture, HEPC is referred to a phospholipid with long hydrocarbon chain length and high phase transition temperature of 50–55°C; on the contrary, the other (naturally occurring EPC) containing high content of unsaturated fatty acid chains is considered to have a lower phase transition temperature of −8°C. The difference of phase transition temperatures between the two PCs is estimated to 60°C at least. It could be speculated that the separated phases, a gel phase and fluid (liquid-crystal) phase, on bilayer membrane at a given temperature were formed, like the giant unilamellar liposomes made of DPPC and DLPC and visualized by confocal microscope [22, 30]. Moreover, gel-gel [31, 32] and fluid-fluid [33] demixing of the binary phospholipid system have also been observed, especially when their hydrocarbon chain lengths are mismatched. Therefore, a combination of two phospholipids including HEPC and natural EPC is reasonably expected to produce liposomes with the many segregated microdomains coexisting on the membrane. 

 Accordingly, formation of the phase boundary was speculated to restrict the lateral diffusion across segregated domains, hindering the self-aggregation of hydrophobic molecules. A stable liposome formulation able to incorporate a high content of paclitaxel, therefore, can be made. The coexistence of lateral separate phospholipid regions promotes the incorporation of a large amount of hydrophobic paclitaxel into the phospholipid bilayer. The hypothesis may account for why the liposomes formulated in this study can incorporate more paclitaxel and remain more stable in long-term storage. The drug to phospholipid molar ratio can be increased to 15%, which was significantly upgraded by approximately sixfolds in comparison to the other liposome formulations reported [12–15, 19]. The liposomes consisting of a combination of two phospholipids showed improved drug loading capacity and shelf stability over those of the formulations with single phospholipid alone. The features even are superior to the previous liposome formulations with negatively charged phospholipids [11–14, 21]. Furthermore, the liposomes still alleviate acute toxicity without changing its cytotoxicity against tumor cells, resembling the other liposome formulations [2, 14, 21, 34, 35]. Pharmacokinetic data also exhibits a higher AUC in rats than Taxol. Despite, the liposomes were not able to circulate in blood as long as those composed of MPEG on the surface. This result may be attributed to the presence of reticuloendothelial (RES) system. Nanoparticles will usually be taken up by the liver, spleen, and other parts of the RES depending on their surface characteristics, especially for particles with more hydrophobic surfaces [36, 37]. However, the RES uptake of liposomal paclitaxel may limit the systemic exposure of non-RES tissues, such as the bone marrow, to paclitaxel [37]. Due to the alternant biodistribution of paclitaxel by liposomes, it may exert not only a direct effect on reduced toxicity but also may underlie the preservation or enhancement of antitumor efficacy following administration of liposomal paclitaxel [37]. Regardless of the similar profile of the blood exposure to Taxol, the prepared liposomal paclitaxel did demonstrate the reduced toxicity and increase the efficacy against human cancer in animal model.

## 5. Conclusion

 This study presents a novel liposomal formulation capable of incorporating a high paclitaxel content, and remaining stable in long-term storage as well. Liposomes remained stable in liquid form at 4°C for at least 6 months when the drug-to-lipid molar ratio was below 15%. In aspects of *in vitro* and *in vivo* efficacy studies, the paclitaxel liposomes exhibit a comparable cytotoxicity against colon cancer and enhanced efficacy against human lung tumor as compared with Taxol. As expected, the liposomes have lower acute toxicity significantly in mice than the current cremophore/alcohol formulation dose. These results demonstrate that the liposomal paclitaxel is promising as an anticancer treatment. The novel formulation has a potential to incorporate the high content of hydrophobic drug stably.

## Figures and Tables

**Table 1:** Characteristics and shelf stability of liposomes mainly composed of either natural EPC or HEPC alone.

Liposome composition	[Lipid] (mM)	Drug/PL (mole%)	[Paclitaxel] (mg/mL)^a^	Mean particle size (nm)	I.E.^b^ (%)	Remaining content^c^ (%)	
						14 days	30 days
EPC/Chol/MPEG	20	3	0.45	142.9	88.4	89.3	77.9
(20/8/1)	20	7	0.52	174.1	42.1	67.8	35.4
HEPC/Chol/MPEG (20/8/1)	20	3	0.32	93.2	68.1	76.7	63.6

^a^Concentration of paclitaxel at day 0.

^b^Incorporation efficiency = paclitaxel incorporated in liposomes/paclitaxel added.

^c^Remaining content = [paclitaxel] at day N/[paclitaxel] at day 0.

**Table 2:** Paclitaxel incorporation efficiency and particle size of the liposomes made of EPC and HEPC. Liposomes were prepared in accordance to the formulation (paclitaxel/total PC/cholesterol/MPEG = 0.3/10/1/0.5).

Molar ratio of HEPC/total PC (%)	Mean particle size (nm)	I.E. (%)
25	113.3	69.2
43	120.8	63.8
62	128.4	73.6
81	202.6	37.6

**Table 3:** Effects of increasing paclitaxel to lipid molar ratio on physical properties of liposomes. Liposome formulation is composed of PCs, cholesterol, and MPEG at the optimal molar ratio (EPC/HEPC/Chol/MPEG = 15/5/2/1).

PC (mM)	Drug/PL^a^ (mole%)	[Paclitaxel] (mg/mL)	Mean particle size (nm)	I.E. (%)
20	7	1.0	114.3	84.5
40	7	2.0	115.8	82.4
20	10	1.3	116.2	78.8
20	15	2.1	125.4	81.0
20	20	2.9	134.9	85.1
20	25	2.3	146.3	54.6

^a^PL represents total phospholipids including EPC, HEPC and MPEG.

**Table 4:** Mean pharmacokinetic parameters of paclitaxel after i.v. injections of Taxol or paclitaxel liposomes at a dose of 5 mg/kg in rats.

	*T*_1/2_ (hour)	AUC_0→*∞*_ (*μ*g h/mL)
Taxol	2.4 ± 0.4	8.5 ± 1.7
Liposomal paclitaxel	2.1 ± 1.0	12.7 ± 5.8

**Table 5:** Survival rate of mice received i.v. injections of Taxol or paclitaxel liposomes at doses of 20 and 40 mg/kg.

	Dose (mg/kg)	Survival Rate
Taxol	20	4/5
	40	1/5
Liposomal paclitaxel	40	8/8
